# Brachial Plexus Anatomy: Normal and Variant

**DOI:** 10.1100/tsw.2009.39

**Published:** 2009-04-28

**Authors:** Steven L. Orebaugh, Brian A. Williams

**Affiliations:** University of Pittsburgh, UPMC South Side, Pittsburgh, PA 15203, USA

**Keywords:** brachial plexus, anatomy, imaging, nerve blockade, ultrasound

## Abstract

Effective brachial plexus blockade requires a thorough understanding of the anatomy of the plexus, as well as an appreciation of anatomic variations that may occur. This review summarizes relevant anatomy of the plexus, along with variations and anomalies that may affect nerve blocks conducted at these levels. The Medline, Cochrane Library, and PubMed electronic databases were searched in order to compile reports related to the anatomy of the brachial plexus using the following free terms: "brachial plexus", "median nerve", "ulnar nerve", "radial nerve", "axillary nerve", and "musculocutanous nerve". Each of these was then paired with the MESH terms "anatomy", "nerve block", "anomaly", "variation", and "ultrasound". Resulting articles were hand searched for additional relevant literature. A total of 68 searches were conducted, with a total of 377 possible articles for inclusion. Of these, 57 were found to provide substantive information for this review. The normal anatomy of the brachial plexus is briefly reviewed, with an emphasis on those features revealed by use of imaging technologies. Anomalies of the anatomy that might affect the conduct of the various brachial plexus blocks are noted. Brachial plexus blockade has been effectively utilized as a component of anesthesia for upper extremity surgery for a century. Over that period, our understanding of anatomy and its variations has improved significantly. The ability to explore anatomy at the bedside, with real-time ultrasonography, has improved our appreciation of brachial plexus anatomy as well.

## INTRODUCTION

When compared with general anesthesia for surgery of the upper extremity, brachial plexus blockade has many potential benefits, including superior pain control, reduced requirement for systemic opioids, improved range of motion after various procedures, and facilitation of early discharge after ambulatory procedures. Effective analgesia via this route requires an understanding of the anatomy of the plexus. This review addresses upper extremity anatomy pertinent to the conduct of brachial plexus nerve blockade, as well as related anatomic variations, in an attempt to improve the anesthesiologist's ability to guide needle to nerve safely.

## METHODS

This review article is based on data related to upper extremity peripheral nerve anatomy that have been featured in the surgery, anesthesiology, and anatomy literature. The Medline, Cochrane Library, and PubMed electronic databases were searched in order to compile case reports, review articles, and clinical and laboratory research reports related to the anatomy of the brachial plexus using the following free terms: "brachial plexus", "median nerve", "ulnar nerve", "radial nerve", "axillary nerve", and "musculocutaneous nerve". Each of these topics was then paired with the MESH terms "anatomy", "nerve block", "anomaly", "variation", and "ultrasound". The following limits were applied: Studies with abstracts; Humans only; Published in Core Clinical Journals. The electronic search was also expanded to include related articles. Resulting articles were hand searched in order to obtain relevant literature that characterizes anatomic positions and anomalous courses of peripheral nerves and surrounding structures. When possible, quantitative data regarding anatomic relationships of nerves to nearby structures were extracted. Only articles that could impact the regional anesthesiologist's understanding of anatomy related to brachial plexus nerve blockade were included. A total of 68 searches were conducted, with a total of 377 articles obtained that met the above criteria. Of these, a total of 57 were found to provide useful information related to the goals of this review.

## RESULTS AND REVIEW

### The Brachial Plexus and its Fascial Investment

Most data related to variations from the normal plexus structure derive from anatomic dissections. The classic description of the plexus, with the C5 to T1 nerve roots combining to form three trunks, six divisions, three cords, and five main motor/sensory branches to the upper extremity, is an oversimplification to some degree. In actuality, many variant forms of the brachial plexus exist, with none representing a majority of patients. Kerr catalogued 29 forms of the brachial plexus among some 175 cadaver specimens dissected between 1895 and 1910[1]. In the early part of the last century, one author described a total of 38 variations of the plexus[2]. Up to 53.5% of plexuses in cadaver studies possess significant anatomic variation from the "classic" description of the brachial plexus[3,4].

The nerve roots of the plexus, as they exit the intervertebral foramina, are typically comprised of a single large fascicle, surrounded by a scant amount of epineurium[3]. These then divide and recombine along the length of the plexus until, eventually, most fascicles have been segregated into largely motor or sensory axons, bound for a particular area or structure[5]. It is clear that the number of fascicles increases and their cross-sectional area decreases, from proximal to distal in the plexus. At the same time, the amount of connective tissue of the epineurium progressively increases from proximal to distal[3,6].

High-fidelity ultrasonography has permitted appreciation of fine anatomic details of the plexus, such as the trunks, divisions, and cords, as well as appreciation of anatomic variability. Demondion et al. mapped the brachial plexus with sonography in 12 volunteers[7]. In the interscalene groove, with the transducer in a parasagittal position, the trunks appear as hypoechoic ovals, cephalad and posterior to the subclavian artery. At this level, the C8 and T1 roots were difficult to identify because of their depth. The authors noted that the deep cervical artery, a branch of the costocervical artery, was frequently identified as it coursed posteriorly between the C7 and C8 nerve roots. In the supraclavicular area, the divisions of the plexus are evident superior and posterior to the subclavian artery[8]. In addition, the transverse cervical artery is often seen with ultrasound imaging, branching from the thyrocervical trunk (a branch of the subclavian artery) and coursing laterally in the suprclavicular area (Fig. 1). This vessel may be very close to, or even lie between, the trunks or divisions of the brachial plexus[9]. Distal to the clavicle, the elements of the plexus are also well visualized with ultrasound, as will be described.

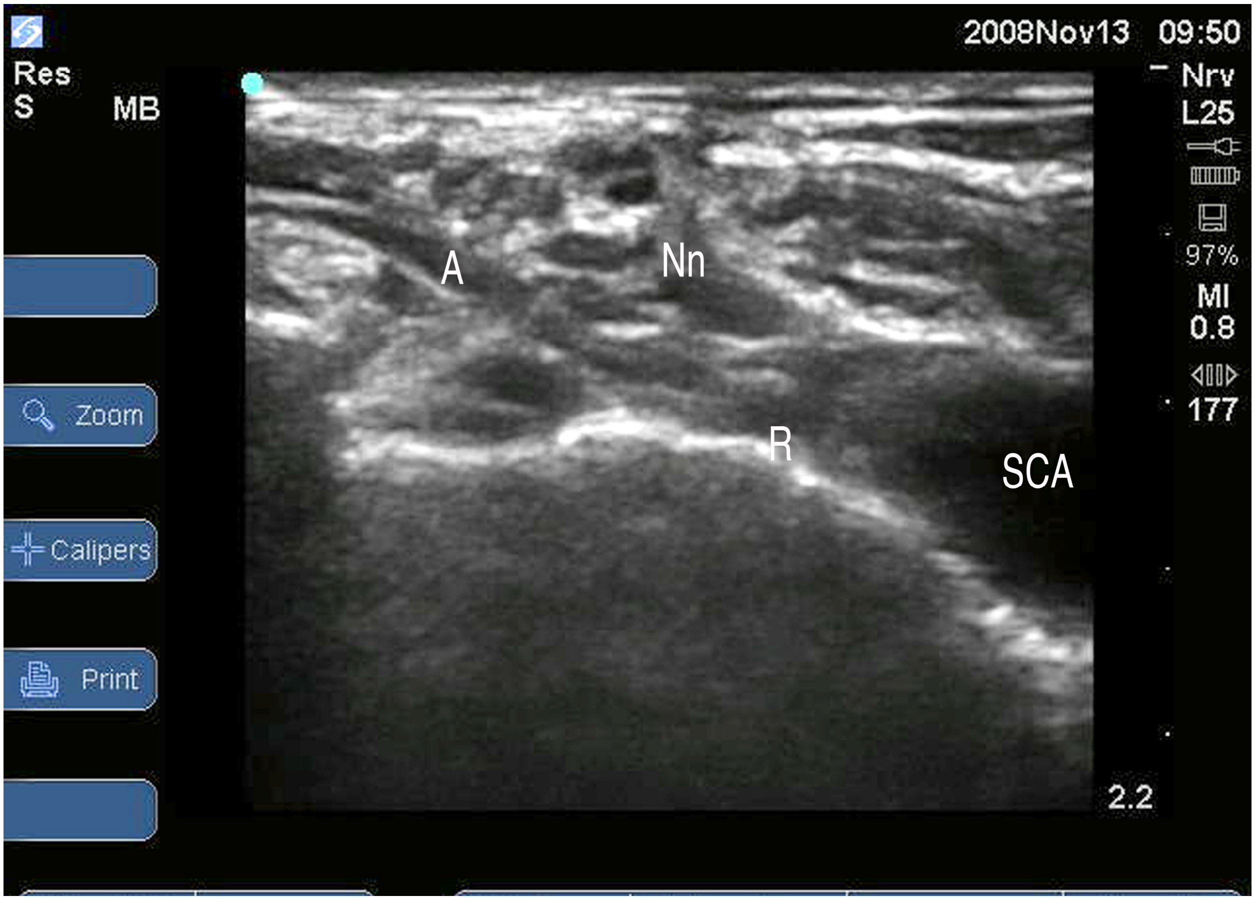

**FIGURE 1.** Ultrasound image of the supraclavicular area, revealing a branch of the subclavian artery coursing between the elements of the brachial plexus. SCA, subclavian artery; Nn, nerves of brachial plexus; R, first rib; A, branch of subclavian artery, probably the transverse cervical artery.

The existence of a “sheath” of connective tissue investing the plexus and its accompanying vessels, segregating this neurovascular bundle from surrounding tissues, continues to be a source of some debate[10,11,12]. While some cadaver dissection studies have suggested the presence of a sheath, radiographic studies utilizing differing modalities have been less supportive[13,14]. Despite earlier assertions to the contrary, the investing connective tissues of the brachial plexus do not form a simple, continuous, tubular sheath. The filmy, septated, and convoluted nature of these investing connective tissues constrains free circumferential flow of contrast or local anesthetic around nerves and vessels; instead, the sheath may sequester, trap, and confine injected solutions. This may impact on the quality of peripheral nerve blockade, especially at more distal levels.

## Anatomy of the Interscalene Region

At the level of the interscalene groove, the “envelope” provided by the scalene muscles and their investing fascia provides a well-demarcated potential space into which to inject local anesthetic solutions, producing brachial plexus anesthesia[15]. While this is regarded as a “brachial plexus block”, the anatomy of this area provides an important constraint. Due to its position between the subclavian artery and the middle scalene muscle, significantly inferior to the point at which the needle is introduced, the inferior trunk is usually not completely anesthetized by this technique, producing the ulnar sparing that is typical of blockade at this level[16,17].

The interscalene groove lies posterior to the clavicular head of the sternocleidomastoid muscle. Most regional anesthesia atlases suggest that the most superficial elements of the brachial plexus should not be deeper from the skin surface than 1–2 cm at this level[16,17]. In a sonographic assessment of the anatomy of the brachial plexus, Yang et al. reported an even shallower average depth from skin to plexus of 5.5 mm[8]. A relationship of plexus depth to body mass index (or body habitus) has not been established. Axial CT or MR imaging at the level of the interscalene groove reveals the close proximity of vulnerable structures to the target nerves here, including the vertebral artery, dural nerve root sheaths, and the neuraxis[18,19].

Use of real-time ultrasonography has become increasingly affordable, practical, and detailed in its discrimination of neural elements, facilitating nerve blocks at the interscalene level in patients with challenging anatomy, for whom nerve stimulator-guided interscalene blocks (ISB) are unsuccessful[20]. At the level of the ISB, in the axial oblique plane of section, the hypoechoic fascicles of the nerve trunks can be visualized passing between the scalene muscles, which are also somewhat hypoechoic (Fig. 2)[21]. Nearby vascular structures, including the internal jugular vein and carotid artery, are well visualized.

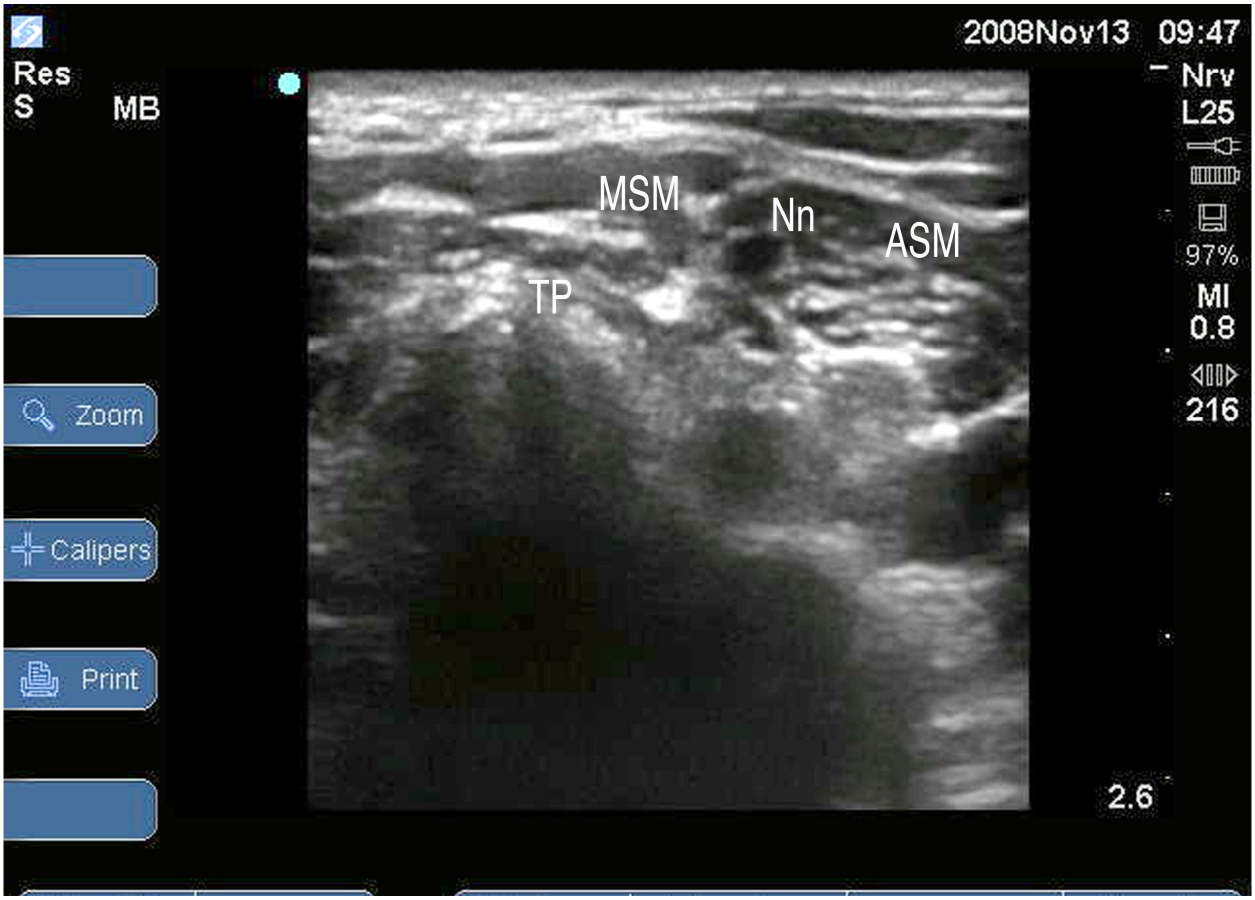

**FIGURE 2.** Ultrasound of the interscalene region, revealing the hypoechoic, nodular fascicles of the nerves of the brachial plexus, between the scalene muscles. MSM, middle scalene muscle; ASM, anterior scalene muscle; Nn, fascicles of brachial plexus nerves; TP, transverse process of cervical vertebra.

The scalene muscles, and the sometimes-elusive groove between them, are the chief landmarks for successful localization of the brachial plexus in ISB. However, the relationship between roots, trunks, and muscles is variable. In a study of 51 cadaver specimens, Harry et al. found that only 32% had the classic anatomic arrangement of the scalene muscles and the brachial plexus bilaterally in the neck[22]. The most frequent abnormality involved the C5 and/or C6 nerve roots passing either through or anterior to the anterior scalene muscle, instead of through the interscalene groove. When this occurs, it is possible that the aberrant nerve roots may confound localization of the plexus with the nerve stimulator. Natsis et al. described brachial plexus anomalies in 93 cadaver dissections and found that 25% had an abnormal superior trunk, the most common example of which was the trunk perforating or running anterior to the anterior scalene muscle[23].

Kessler and Gray were able to visualize this anomaly in volunteers in which the plexus and its surrounding anatomy were imaged in the interscalene region with ultrasonography[24]. In 13% of these volunteers, variations from the typical relationship of the scalene muscles and brachial plexus roots were present, the most common being the C5 nerve root running through or outside the anterior scalene muscle. The authors postulate that this arrangement may be responsible for occasional incomplete interscalene blocks.

The plexus may lie more cephalad or caudad than typical in different individuals, affecting the performance of ISB. In a study of 200 brachial plexus specimens in spontaneously aborted fetuses, Uysal et al. found that 46.5% of the specimens possessed classic organization, while 53.5% possessed some degree of variation from the accepted norm[4]. One-fourth of the plexus specimens were of the "prefixed" type, in which the C4 root joins the plexus, while 2.5% were of the "postfixed" type, with a contribution from T2 root. Bonnel earlier described the "prefixed" and "postfixed" subtypes in adult specimens, finding 41 of 100 being "prefixed" and four of 100 being "postfixed"[3]. Occasionally, the superior trunk does not form (i.e., the roots of C5 and C6 remain distinct)[3].

Furthermore, other nerves used to help localize the brachial plexus during ISB may be aberrant in their course. The phrenic nerve, when stimulated, typically prompts the operator to move the needle tip in a posterior direction, as this nerve typically courses along the anterior scalene muscle as it descends the neck[17]. However, an accessory phrenic nerve is a common occurrence, occurring in up to 75% of patients[2]. The accessory phrenic nerve usually runs with the nerve branch to the subclavius muscle, arising from the superior trunk. Stimulation of the accessory phrenic nerve may mislead the operator in his interpretation of the local anatomy. Another described anomaly that may have similar consequences is the phrenic nerve arising entirely from the brachial plexus[25].

To evaluate the existence of a brachial plexus "sheath" at proximal levels, Yang et al. inserted catheters into the interscalene groove under real-time ultrasound guidance[8]. Injection of contrast under fluoroscopy was used to confirm catheter position, and the movement of contrast was studied as evidence of the conformation and integrity of the sheath enveloping the brachial plexus at this region. Injected contrast collected in the interscalene groove, but also made its way out of the apparent fascial confines of this space, enveloping the scalene muscles in 90% of subjects and abutting the carotid sheath in half. The authors concluded that the connective tissue sheath surrounding the brachial plexus is discontinuous.

## Anatomy of the Supraclavicular Region

Medial to the clavicle, the scalene muscles descend to the first rib, onto which they attach. The subclavian artery and the brachial plexus emerge from the interscalene groove, deep to the supraclavicular fossa, and course laterally and inferiorly to pass under the clavicle. The brachial plexus at this level is comprised of a compact group of six divisions, which is perhaps the portion of the plexus most anatomically favorable for complete blockade of the plexus with a single injection.

Cornish and Leaper examined the anatomy of the brachial plexus "sheath" utilizing CT scanning with indwelling supraclavicular nerve block (SCB) catheters at this level of the plexus[13]. They compared the CT dye studies of this region in patients with those of patients with indwelling sciatic nerve block catheters and found that those of the upper extremity resembled those of the lower extremity in that the limits of dye spread were defined by surrounding anatomic structures, not a "sheath" of connective tissue.

The nerve plexus at this level is critically close to the dome of the lung. This increases the risk of needle contact with the pleura or lung, which may result in pneumothorax[26]. Appreciation of this anatomic relationship with real-time ultrasound scanning may reduce this risk and improve the safety of blocks conducted in the supraclavicular region[27].

In order to quantify the relationships of the plexus to surrounding structures, Apan et al. utilized sonography in 30 patients, along with coronal MR images in a subset of these, to examine the supraclavicular region[28]. The authors found that the depth of the brachial plexus from the skin at this level, with the transducer in the coronal oblique position, averaged 1.65 cm in males and 1.45 cm in females.

At the bedside, ultrasound guidance may be used to reveal a transverse view of the divisions of the brachial plexus that manifest as hypoechoic nodules of varying size, lateral and cephalad to the subclavian artery, which itself lies on top of the echogenic first rib. The appearance of the plexus with ultrasound imaging at this location has been likened to a "cluster of grapes" (Fig. 3)[21]. The inferior trunk and its divisions tend to lie very caudad in the groove, often directly on the first rib. As with blocks performed at the interscalene level, this could result in sparing of the contributions from the C8 and T1 nerve roots, unless the inferior trunk is considered. Taking advantage of the opportunity to evaluate the anatomy at the bedside with ultrasound, some authors recommend that a local anesthetic solution be injected in close proximity to these nerve elements, just posterior to the artery, causing the nerves to float upwards in the injected liquid[29].

## Anatomy of the Infraclavicular Region

Beneath the clavicle, the cervicoaxillary canal is formed, bounded by the first rib below and the clavicle above. Through this passageway, the vessels and brachial plexus enter the apex of the axilla. Overlying this region is the infraclavicular fossa. Deep to the skin are the pectoralis major and minor muscles. Posterior to the neurovascular bundle at this region lie the serratus anterior muscle and, posteromedially, the pleura. The anatomic advantage of approaching the plexus in this region is that it appears to pose less risk of pneumothorax than with the supraclavicular approach.

The consequences of shifting brachial plexus blockade laterally is a reduced likelihood of blocking the entire plexus with a single injection, since the terminal branches begin to leave the cords and course toward their respective targets at about the level of the coracoid process, or even more proximally. Since both the axillary and musculocutaneous nerves usually leave the plexus shortly after the formation of the cords from which they originate, these may be more difficult to anesthetize at this level.

Imaging has been utilized to good advantage to investigate anatomic relationships relative to the infraclavicular brachial plexus. Raphael et al. utilized MRI in multiple volunteers to create a "frontal slab" projection, which represents a three-dimensional composite image[30]. While not practical as a bedside technique, this allows both a high-definition examination of the plexus and comparison of various block techniques. MRI was also used in a study of volunteers by Sauter et al. to evaluate the position of the cords around the axillary artery in 10 volunteer patients[31]. The authors found that the cords consistently lay within 2.5 cm of the center of the artery, in a range from directly inferior to cephaloanterior, arranged circumferentially around the vessel.

High-resolution sonography may be particularly useful in delineating the anatomy of the infraclavicular region. The hypoechoic ovals apparent at more proximal regions (either ISB or SCB) are not apparent below the clavicle; instead, the cords of the plexus appear to be hyperechoic, most likely due to increasing amounts of connective tissue within the epineurium as the plexus travels distally[3]. In transverse section, the cords are typically arrayed superior (lateral cord), inferior (medial cord), and posterior to the axillary artery (posterior cord)[32] (Fig. 4).

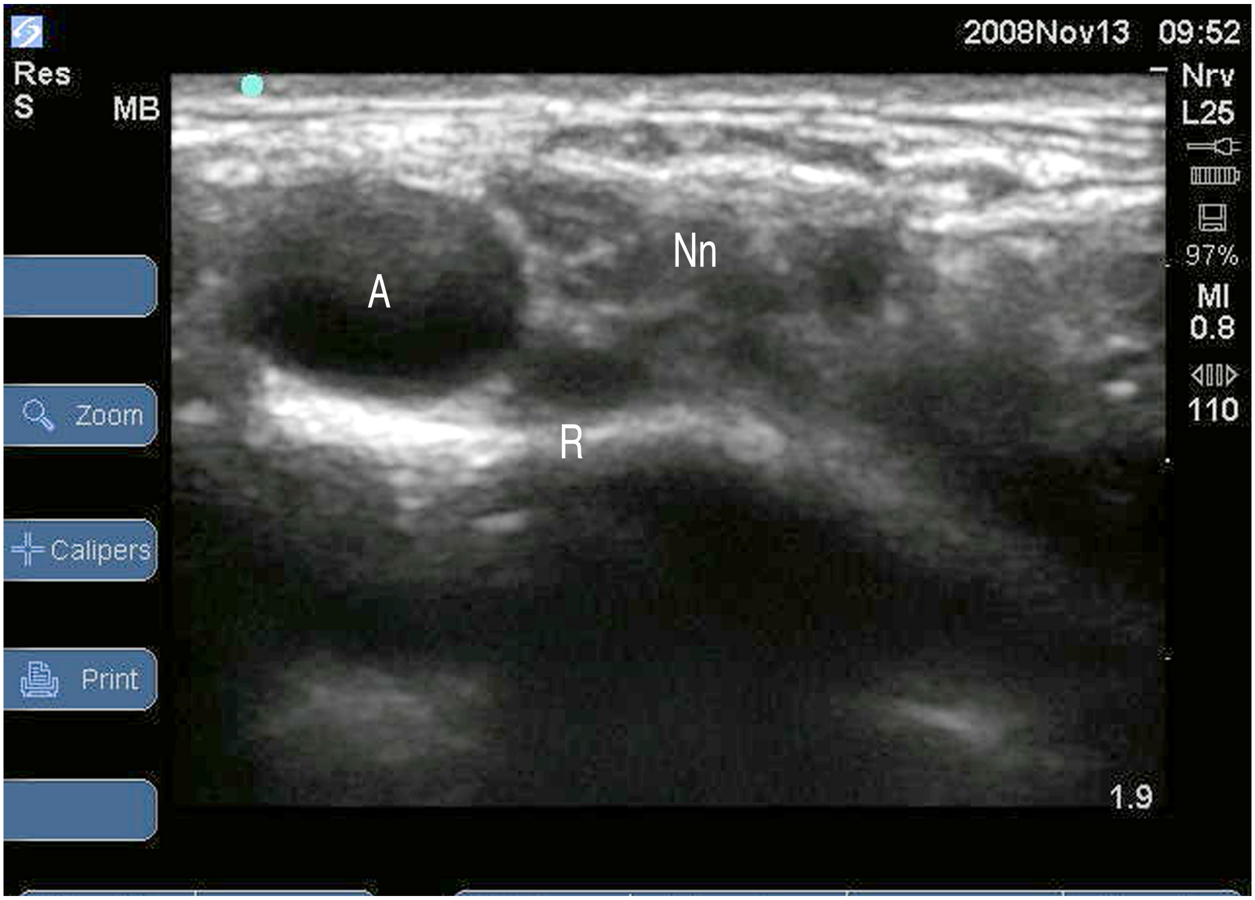

**FIGURE 3.** Ultrasound scan of supraclavicular region, revealing the nerve elements of the brachial plexus, clustered lateral to the artery. A, subclavian artery; R, first rib; Nn, nerves of brachial plexus.

As noted, the brachial plexus may contain significant variations in the formation of trunks and cords. In 2.5–4% of cases, the T2 root contributes to the plexus[3,4]. In many specimens, no inferior trunk exists[4]. Likewise, it is common for no discrete posterior cord to form; the posterior divisions instead diverge to give rise to the terminal nerves and branches directly[3,33]. Other described variations in the plexus include failure of T1 to contribute to the posterior cord; also, the T1 root may be the only contributor to the medial cord. A pair of cords, one large and one small, may replace the usual pattern of three cords[2]. Cases of formation of a single cord have been noted[34]. Anomalies of the distal brachial plexus, as well as the axillary vessels, could prove problematic during attempted nerve block at the infraclavicular level. Real-time use of ultrasound may contribute to better understanding and more successful blockade in individual cases.

Anomalous arteries and veins in the infraclavicular region may increase the risk for intravascular injection. The axillary artery may be formed into two parallel arteries that arise from the subclavian artery, and then course distally as the ulnar and radial arteries[2]. The subclavian artery may also give rise to branches, such as the circumflex humeral, the circumflex scapular, and thoracodorsal arteries, which normally arise more distally.

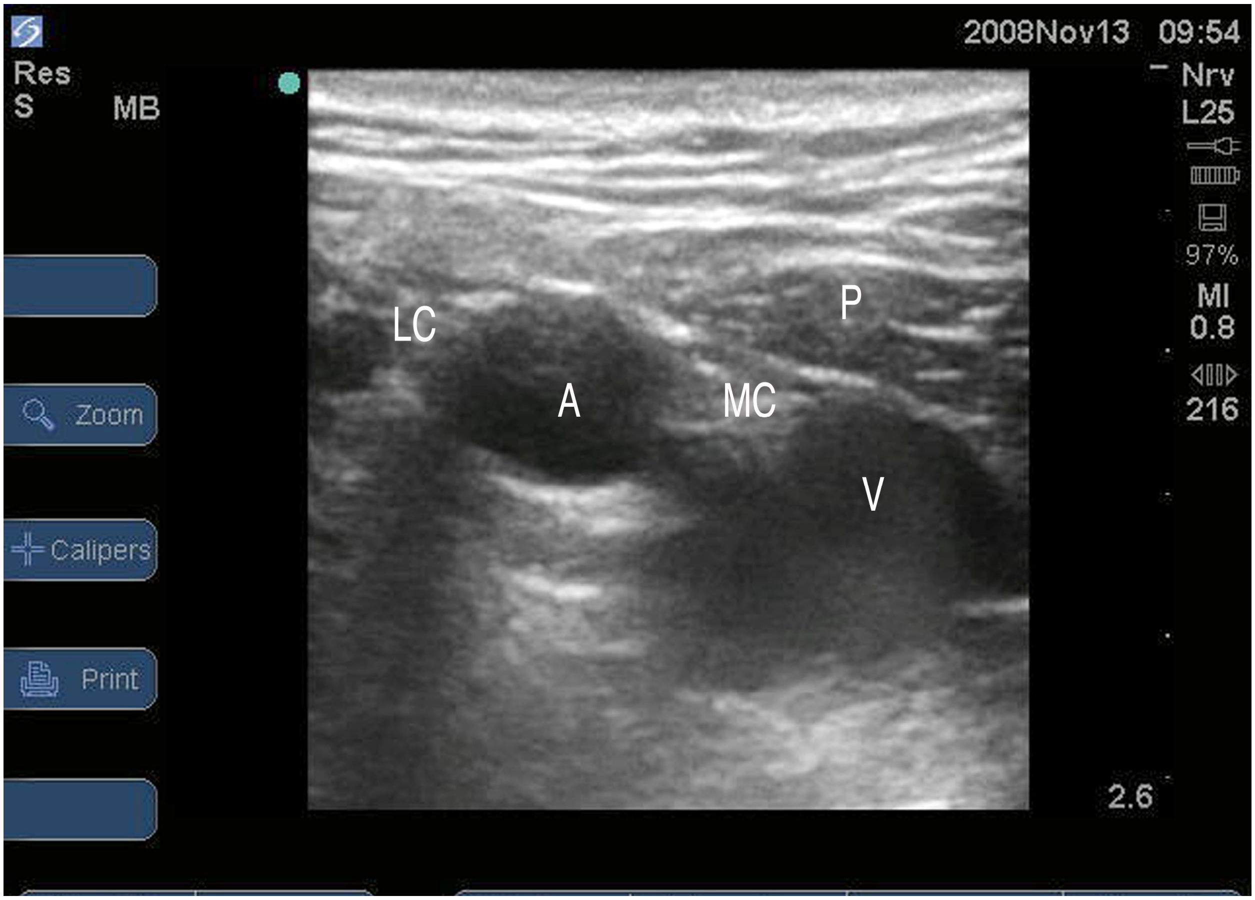

**FIGURE 4.** Ultrasound image from infraclavicular region, showing the cords of the brachial plexus with the axillary artery and vein. The posterior cord is not well imaged and probably lies directly posterior to the artery. A, axillary artery; V, axillary vein; P, pectoralis minor muscle; LC, lateral cord of brachial plexus; MC, medial cord of brachial plexus.

## Axillary Block

The axilla is a pyramidal space beneath the glenohumeral joint that serves as a conduit for vessels and nerves to reach the upper extremity. Its anterior wall is formed by the pectoralis muscles. The posterior wall is made up of the subscapularis muscle superiorly, and the teres major and latissimus dorsi muscles inferiorly. Medially are the chest wall and serratus anterior muscles, while laterally lie the humerus and its muscular attachments[16]. The axillary nerve and musculocutaneous nerve emerge from the posterior and lateral cords, respectively, proximally in the axilla, rendering unlikely the potential for anesthesia of the entire brachial plexus with a single injection of local anesthetic at this level[35].

As with approaches at more proximal levels, a connective tissue sheath of the brachial plexus at axillary levels is present, but variable, in its degree of neural investment and compartmentalization. In a clinical MRI study of the spread of local anesthetic inside the axillary sheath, Klaasted et al. inserted single-lumen catheters into the sheath[14]. A series of gradually increasing volumes of contrast-containing local anesthetic was injected via the catheter, and the spread of the solution and quality of the resultant nerve block were evaluated. Eight of 13 (62%) subjects had incomplete spread of contrast circumferentially about the artery on MRI scan; the remaining five of 13 (38%) had complete circumferential filling, with clear improvement in the quality and extent of nerve blockade of the radial, ulnar, and median nerves, when compared with subjects who had incomplete filling.

Textbooks commonly describe a pattern of terminal nerve position in the axillary neurovascular bundle when the arm is abducted 90 degrees at the shoulder, with the median nerve lateral to the artery, the ulnar nerve anteromedial, and the radial nerve posterior to the brachial artery[16,17]. *In vivo* studies with ultrasonography support this pattern, but also reveal significant variations in the position of these nerves. Retzl et al. reported that, in a series of 69 volunteers, the ulnar nerve was anteromedia to the axillary artery in 59% of cases. The radial nerve was positioned medial to posteromedial to the vessel in about two-thirds of cases, while the median nerve was anterolateral or anteromedial to the vessel in 56% (Fig. 5)[36]. Surprisingly, the authors found that these nerves changed position readily, with even mild pressure on the skin. Similarly, Chan et al., in a study comparing ultrasound-guided axillary block to nerve stimulator-guided block, reported that the median nerve was usually anterolateral to the brachial artery, the ulnar nerve anteromedial, and the radial nerve posterior or posteromedial[37].

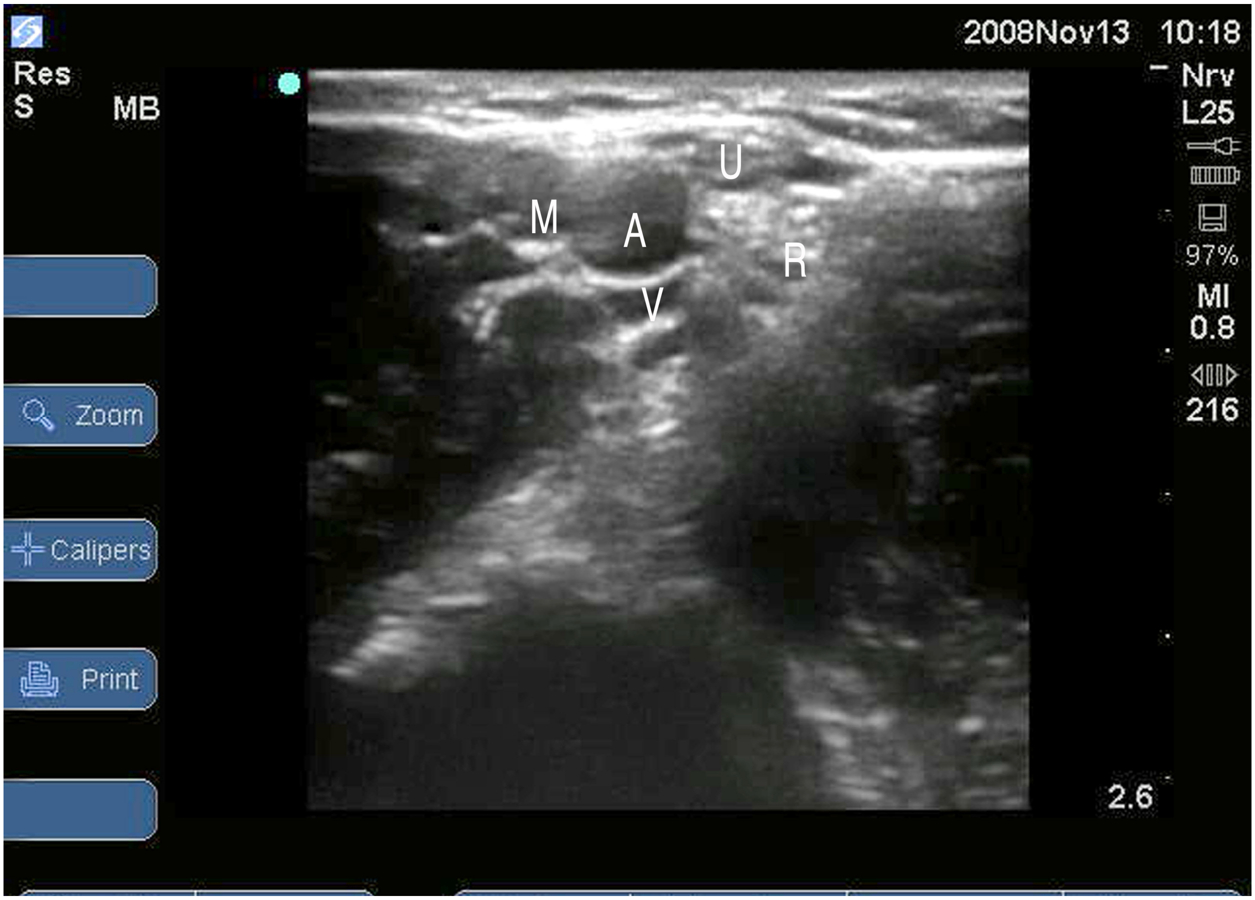

**FIGURE 5.** Ultrasound image of axillary region, showing nerves in neurovascular sheath. A, axillary artery; V, compressed axillary vein; M, median nerve; U, ulnar nerve; R, radial nerve.

Neither the median nerve nor the ulnar nerve normally branches in the axilla. However, the radial nerve, which innervates the dorsal musculature of the arm as well as the forearm, begins its branching in the axilla. In attempting to stimulate the radial nerve during axillary block selectively, a range of responses can be expected, including finger or wrist extension, supination, or elbow extension. Because the initial branches of the radial nerve, those to the long head of the triceps, leave the nerve in the proximal axilla[38], delivering local anesthetic when only triceps stimulation is apparent may block only these branches, rather than the entire radial nerve. Despite this anatomic concern, clinical studies have not shown a higher failure rate of radial nerve block when distal (wrist or fingers) vs. proximal (triceps) motor twitches are accepted[39].

The musculocutaneous nerve (MCN) is most often found outside the neurovascular bundle at axillary levels. This nerve typically runs between the two heads of the coracobrachialis muscle. Ultrasound has been utilized to show its characteristic position and shape as it courses down the arm[40]. Its shape varies from elliptical to flattened, and it moves progressively further away from the artery as it proceeds distally, moving into a plane between the biceps and brachialis muscles, both of which it innervates.

The medial brachial cutaneous (MBC) and medial antebrachial cutaneous (MABC) nerves innervate the skin of the medial arm and forearm, respectively. Both arise from the medial cord, although the MABC may branch directly from the inferior trunk. The course of the MBC generally parallels the ulnar nerve as it moves down the medial aspect of the arm. It quickly begins to branch to innervate the skin of the medial arm. In 90% of cases, this nerve communicates with the intercostobrachial nerve, which has its origin from the second intercostal nerve. Two to three centimeters proximal to the medial epicondyle, the MBC gives off two to five terminal branches[41]. In most people, the branches of the MBC terminate in the skin over the medial epicondyle.

The MABC nerve courses distally, parallel to the ulnar nerve, lying lateral to it and medial to the brachial artery. This nerve emerges from the deep brachial fascia at the middle of the arm to run with the basilic vein, crossing it. Often, the MABC provides a branch to the medial aspect of the distal arm, and approximately 2 cm proximal to the medial epicondyle divides into five to eight branches to supply the skin over the olecranon and the ulnar aspect of the forearm[41,42]. In 10% of cases, the MABC communicates with the MBC or the ulnar nerve. Authors differ with regard to elbow joint innervation by the MABC. Masear et al. found articular branches to the elbow in one-third of 50 cadaver specimens[42].

Variations of the relationship of the brachial plexus to the axillary artery in the axilla, and to the brachial artery more distally, were noted by Miller to occur in 8% of 480 dissections, including the artery lying superficial to the median nerve, division of the median nerve or a cord by branches of the artery, and changes in the structure of the plexus caused by an aberrant axillary artery or branch[43]. Uysal et al. described cases in which the two roots of the median nerve did not join until they reached the level of the distal arm[4].

Abnormal vascular formations in the axilla are common and may complicate axillary blockade. Kutiyanawala et al. reported that 21 of 100 patients undergoing axillary dissection had abnormal venous architecture in this region, including 10 patients with double axillary veins[44]. Variations in arterial formation are also frequent in this region. Uglietta and Kadir evaluated anomalies in arteriograms of the upper extremity of 100 patients[45]. Nine percent of these cases were remarkable for variant arterial anatomy of the upper extremity, the most common example of which being a high origin of the radial artery, arising from the brachial artery in the arm. Variations with double brachial artery, bifid brachial artery, or a superficial and deep brachial artery are reported[46,47]. Such arterial variations occur in up to 10% of specimens[46]. Variations and anomalies of the ulnar and radial arterial system are more frequent, but usually occur more distally in the arm; therefore, these are not usually relevant to the performance of an axillary block. However, anomalies of the ulnar[48] and radial[49] arteries have been reported to arise high in the arm, on occasion, with possible impact on axillary blockade.

Abnormalities of the neural elements in the axilla are also frequently reported, with the MCN affected most commonly. Prasada Rao and Chaudhary found that the MCN was entirely absent in two (8%) of 24 cadaver dissections, with the innervation of the biceps and brachialis provided by branches of the median nerve[50]. In addition, the MCN may fuse to, or accompany, the median nerve, running distally for a variable distance before separating from it, rather than coursing into the coracobrachialis muscle in the axilla[2]. This has been reported during a case of ultrasound-guided axillary block, in which the MCN was not evident in the coracobrachialis muscle[51].

A second abnormality of the MCN distribution is its communication with the median nerve, either in the axilla or more distally in the arm. In a dissection of 79 cadavers, Venieratos and Anagnostopoulou described unilateral or bilateral connections between the musculocutaneous and median nerves in 20% of patients[52]. In about half of these, the communication occurred prior to the MCN entering the coracobrachialis muscle, and in the others, the MCN-median communication occurred below the coracobrachialis muscle. Loukas and Aqueelah described such communications in 63% of 129 cadavers[53].

## High Humeral Block

Performance of nerve blocks at the high humeral position demands thorough understanding of neural anatomy in the upper extremity, since the four main terminal branches of the brachial plexus, by this point, have begun to disperse and are no longer tightly entwined about the brachial artery in a neurovascular bundle. Dupre described this anatomy and block technique for surgery of the hand and forearm, relying on stimulation of all four of the terminal branches at the junction of the proximal and middle-third of the humerus[54].

The median nerve descends from the axilla in close proximity to the brachial artery, frequently lying just lateral to the artery at high humeral levels. Likewise, the ulnar nerve remains close to the artery to this point and is found primarily just medial to the artery. The radial nerve leaves the axilla posterior to the artery and passes deep to the long head of the triceps, proceeding distally around the humerus in the spiral groove[38]. Bono et al. evaluated the relationships of the radial nerve in this region[55] and found that the branches to the long head of the triceps have usually been released from the radial nerve at this distal position. However, the majority of branches to the medial head and lateral head of the triceps arise at this level, in the brachioaxillary angle and in the spiral groove of the humerus[38].

The MCN at the high humeral level has commonly traversed the coracobrachialis muscle, and runs in a plane between the brachialis and biceps muscles, for both of which it provides innervation.[40]. The nerve may provide one or multiple branches to each of these muscles.

Variations and anomalies of the upper extremity that may impact on the performance of the high humeral block include abnormalities of muscles, vessels, and nerves. As noted in the section on axillary block, double brachial arteries and other anomalies with multiple vessels present in the intermuscular septum may predispose to intravascular injection of local anesthetic. Major variations of the brachial artery are present in about 25% of subjects studied[2]. A superficial brachial artery, lying anterior to the median nerve, occurs in about 13% of anatomy specimens. Nakatani et al. describe bilateral superficial brachial arteries arising from the axillary artery, running lateral to the median nerve down the arm[56]. The biceps may be anomalous, with multiple extra heads, resulting in an aberrant course of either the MCN or the median nerve[57]. It is not uncommon for the median nerve to form from its two roots more distally than usual, in the mid arm[2].

Use of ultrasound at the high humeral level could help to identify the more widely spaced nerves and their depth from the skin surface[21]. The MCN, however, has become smaller, flatter, and further away from the neurovascular bundle at this point, making its detection on ultrasound more challenging[40].

## CONCLUSION

Knowledge of anatomy remains the cornerstone of regional anesthesia. Newer imaging modalities have allowed improved characterization of anatomic relationships for successful nerve blockade in various positions along the brachial plexus and its terminal nerves. Real-time ultrasonography may be particularly useful in characterizing local anatomy, demonstrating nerve positions, and detecting anatomic variation that may affect block success or compromise patient safety.

## ACKNOWLEDGMENTS

The authors acknowledge no conflicts of interest to the preparation of this work. Funding for this project was from departmental resources.

We particularly wish to acknowledge Cristy Paranay for her efforts in the preparation of this manuscript.
